# A full‐length 18S ribosomal DNA metabarcoding approach for determining protist community diversity using Nanopore sequencing

**DOI:** 10.1002/ece3.11232

**Published:** 2024-04-10

**Authors:** Chetan C. Gaonkar, Lisa Campbell

**Affiliations:** ^1^ Department of Oceanography Texas A&M University College Station Texas USA; ^2^ Department of Biology Texas A&M University College Station Texas USA

**Keywords:** ciliates, diatoms, dinoflagellates, microbial diversity, minION, primer design

## Abstract

Protist diversity studies are frequently conducted using DNA metabarcoding methods. Currently, most studies have utilized short read sequences to assess protist diversity. One limitation of using short read sequences is the low resolution of the markers. For better taxonomic resolution longer sequences of the 18S rDNA are required because the full‐length has both conserved and hypervariable regions. In this study, a new primer pair combination was used to amplify the full‐length 18S rDNA and its efficacy was validated with a test community and then validated with field samples. Full‐length sequences obtained with the Nanopore MinION for protist diversity from field samples were compared with Illumina MiSeq V4 and V8‐V9 short reads. Sequences generated from the high‐throughput sequencers are Amplicon Sequence Variants (ASVs). Metabarcoding results show high congruency among the long reads and short reads in taxonomic annotation at the major taxonomic group level; however, not all taxa could be successfully detected from sequences. Based on the criteria of ≥95% similarity and ≥1000 bp query length, 298 genera were identified by all markers in the field samples, 250 (84%) were detected by 18S, while only 226 (76%) by V4 and 213 (71%) by V8‐V9. Of the total 85 dinoflagellate genera observed, 19 genera were not defined by 18S dinoflagellate ASVs compared to only three among the total 52 diatom genera. The discrepancy in this resolution is due to the lack of taxonomically available 18S reference sequences in particular for dinoflagellates. Overall, this preliminary investigation demonstrates that application of the full‐length 18S rDNA approach can be successful in field studies.

## Graphical abstract

A new primer pair combination was used to amplify the full‐length 18S rDNA and its efficacy was validated with a test community. The full‐length sequences obtained with the Nanopore MinION for protist diversity from field samples yielded improved taxonomic resolution when compared with Illumina MiSeq V4 and V8‐V9 short reads.

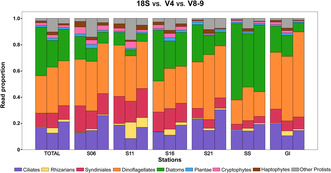

## 1 INTRODUCTION

Phytoplankton are the building blocks of aquatic food webs and comprise a broad spectrum of taxa and functional groups (Cavalier‐Smith, 1993; Litchman et al., 2007). Protist identification is routinely carried out using traditional light microscopy (d'Alcalà et al., 2004); however, the lack of diagnostic characters, scarcity of taxonomic expertise and the time‐consuming nature of these analyses have produced insufficient information on diversity (Barsanti et al., 2021). To overcome these obstacles, DNA based molecular approaches are applied for species identification (Kooistra & Medlin, 1996; Sarno et al., 2007).

In the last decade, environmental DNA metabarcoding has proved to be a powerful molecular‐based approach to assess plankton diversity on both spatial and temporal scales. In metabarcoding, the target DNA sequence is considered a proxy for species detection. An advantage of metabarcoding is the ability to identify unculturable or morphologically indistinguishable taxa. The 18S rDNA gene is one of the most commonly used markers for species identification and delineation due to its conserved and hypervariable regions (V1‐V9) (Kooistra et al., 2010; Medlin et al., 1996). Previous studies of protist diversity using metabarcoding have used one of the hypervariable short read regions in the 18S rDNA e.g., V1‐V2 and V3, V4, V6, V7‐V8, V8‐V9 or V9 (Amaral‐Zettler et al., 2009; Bradley et al., 2016; Bukin et al., 2023; de Vargas et al., 2015; Hadziavdic et al., 2014; Hatteranth‐Lehmann et al., 2019; Massana et al., 2015; Medinger et al., 2010; Mohrbeck et al., 2015; Piredda et al., 2017; Stoeck et al., 2010). Until now, most metabarcoding studies of plankton diversity has been assessed using primer pairs that produce fragments lengths <600 bp. For autotropic protists, the 16S plastidal V4‐V5 region was used (Needham & Fuhrman, 2016), while short reads of nuclear DNA were used for bacteria, archaea, protist and metazoans (Baehren et al., 2023; Curry et al., 2022; Davidov et al., 2020; Lentendu et al., 2022). A short fragment of the ribulose‐1,5‐bisphosphate carboxylase/oxygenase (rbcl) gene was recommended for freshwater diatom diversity (Pérez‐Burillo et al., 2020; Vasselon et al., 2017). The mitochondrial cytochrome oxidase subunit I (COI) was also considered to be one of the standard markers to distinguish closely related species, but there is no universal primer to target the total community (Hamsher et al., 2011). Because all these studies used short reads, the results provide less discriminatory power at the species level (Creer et al., 2016). A primer combination that will provide maximum taxa coverage is needed.

Generally, most studies of protist diversity have focused only on a few of the major eukaryotic domains/subdomains using either the V4 or V9 metabarcode sequences (Piredda et al., 2017; Stoeck et al., 2010; Tragin et al., 2018). The limitation of Illumina MiSeq sequencing is that it can only sequence a maximum of 300 bp using the Illumina MiSeq Reagent Kit v3 (2 × 300 bp). Another concern with using short reads is that rapidly evolving hypervariable regions may introduce more species diversity due to traces of ancient affinities and increase the chances of overestimation of protist diversity (Guo et al., 2022). Selection of a suitable marker for assessing species diversity is crucial for any community study. The selection of appropriate primers for the marker of choice is also critical to accurately depict and assess the entire biodiversity (Alberdi et al., 2018). Unfortunately, there is not a common primer pair for short reads that has a broad taxonomic coverage (Latz et al., 2022; Vaulot et al., 2022).

The advent of long read high throughput sequencers, e.g., Oxford Nanopore Technologies (ONT), enables sequencing of large DNA fragments (Wang et al., 2021). One disadvantage of the long read sequencing technique has been the high error rate compared to Illumina. These errors in Nanopore sequencing are not generated because of the sequencing technique; instead, errors are due to base calling efficiency. The errors are randomly generated, but with the help of self‐correction tools they can be detected and fixed by either mapping with reference sequences, or by using very high accuracy basecalling. At present, ONT has high accuracy basecalling tools that has significantly reduced the error rate, for example, guppy, buttery‐eel, and dorado (Gamaarachchi et al., 2022). Furthermore, Nanopore basecalling has been in continuous development in recent years to improve sequence accuracy (Napieralski & Nowak, 2022; Pagès‐Gallego & de Ridder, 2023). Additional advantages of the ONT include the following: it is cost effective, its small size makes it easily transportable, and the library preparation for sequencing is relatively simple (van der Reis et al., 2023; Wang et al., 2021). By including all the nine conserved and hypervariable regions of the 18S rDNA gene, detailed taxonomic identification (species‐level), even among the closely related species is possible.

In all cases, a major limiting factor for metabarcoding studies is the availability of a well curated taxonomically validated reference database. The Protist Ribosomal Reference database (PR^2^), SILVA, databases are curated and frequently updated with new sequences (Guillou et al., 2012; Quast et al., 2012). Although the 28S rDNA gene has 12 conserved and hypervariable regions with large sequence differences (Zhao et al., 2020), most of the taxonomically curated reference sequences are available only for the D1‐D3 region. This explains why most studies have used the 18S rDNA gene over other markers. By obtaining full‐length 18S rDNA sequences from field samples, this will help to fill the gaps present in the reference sequence database, which will be useful for future metabarcoding studies. Using the phylogenetic approach, a sequence can be defined by aligning it with the most closely associated species.

The aim of this study was to demonstrate the utility of a new primer combination designed for investigations of the eukaryotic microplankton community diversity utilizing the Oxford Nanopore Technologies (ONT) sequencing for targeted long reads using the minION Mk1C. Specifically, the goal was to determine if the full‐length 18S rDNA revealed improved high‐resolution taxonomy and diversity over results from the short read markers V4 or V8‐V9. To examine this question, the results presented here compare the protist diversity obtained by the three markers. To date, no detailed study has been conducted to amplify full‐length 18S rDNA sequences and evaluate the efficiency of this approach to uncover a broad taxonomic coverage of diversity among protists. Given that the objective of this study was to compare species diversity, not abundance, an added beneficial outcome is that these full‐length 18S rDNA sequences from field samples can be used as proxy references in future metabarcoding studies.

## 2 MATERIALS AND METHODS

### 2.1 Samples

#### 2.1.1 Cultures used in the test community for primer validation

Cultures representing each of the major groups of microplankton were used to validate the new primer combination in a test community (Appendix 1). All cultures were grown at 20°C with 100 μmol quanta m^−2^ s^−1^ on a 12:12 light:dark cycle. At mid‐ to late‐exponential phase, cultures were pelleted by centrifugation and stored at −80°C until extracted.

#### 2.1.2 Field samples

Six stations were sampled from Galveston Island near Houston, Texas to south of Port Aransas, Texas (Appendix 2) (Fiorendino et al., 2023). Field surface seawater (500–1000 mL) were filtered onto a 47 mm diameter, 5.0 μm filter (Millipore, USA) in duplicate for DNA extraction. All filters were immediately placed in 2 mL tubes with RNALater (ThermoFisher Scientific, USA) and stored at −80°C until extraction.

### 2.2 Nucleic acid extractions

Nucleic acids were extracted from each filter or culture cell pellet using the AllPrep DNA/RNA MiniKit (Qiagen, USA). DNA was quantified using a Nanodrop (ThermoFisher Scientific Inc, USA). All the gDNA were normalized to 5 ng/μl concentration for amplification.

### 2.3 Primer design and evaluation

#### 2.3.1 Full‐length 18S rDNA primer design

Full‐length 18S rDNA primers were selected based on the available literature: SSUF and SSUR (Medlin et al., 1988). Upon testing these primers with the reference sequences of Chaetocerotaceae, it was revealed that the reverse primer has a site for spliceosomal introns (Gaonkar et al., 2018). These primers were evaluated using in silico test for species detection and its compatibility with different eukaryotic phyla using the 18S rDNA gene. It was evident that the SSUR was not capable of capturing some taxa as the primer site was interrupted with an intron. As an alternative, the primer ITS‐1dr (Edgar & Theriot, 2004) in the 3′ end of the 18S region was evaluated for primer compatibility. The SSUF and ITS‐1dr primer combination was tested using TestPrime against the SILVA database (Quast et al., 2012) with the following parameters: maximum number of mismatches of three bases and zero mismatches at the 3′ end (Klindworth et al., 2013).

#### 2.3.2 Evaluation of the samples for long reads

After in silico validation of the new primer combination, amplifications were performed in duplicate using genomic DNA from each of the six cultures representative of the major protist groups (hereafter, “test community”) to evaluate the efficiency and compatibility of the new combination of the SSUF and ITS‐1dr primers. Cyanobacterial and *Escherichia coli* DNA were used as negative controls to validate the specificity of amplification and a field sample as a positive control. Amplifications were performed in a reaction mixture containing ~5 ng of extracted DNA, 0.5 μM primers, and 1× repliQa HiFi ToughMix (QuantaBio, USA) in a final volume of 25 μL in duplicates. Thermocycler parameters were set at initial denaturation 96°C for 300 s, 35 cycles of denaturation at 96°C for 30s, annealing at 52°C for 30 s, extension at 72°C for 90 s, with a final extension step of 20 min at 72°C. All PCR included a nuclease‐free water negative control. The amplified products were visualized on an agarose gel under UV illumination. All PCR products were cleaned using NEB ExoCIP Rapid PCR Cleanup Kit (NEB, USA) and Sanger sequenced to evaluate the identification of the species used in the test community. On confirming the accuracy of each PCR product, the remainder was used for ONT minION library preparation (See Section 2.4, below). Amplifications were conducted for the field samples with a similar procedure. For each station, duplicate field samples were amplified, each using two separate PCRs; all were pooled to cover maximum species richness.

#### 2.3.3 Evaluation of the test community for short reads

Amplifications were performed using DNA from each of the representative six cultures using the primers for V4 using Reuk454FWD1 and ReukREV3 (Stoeck et al., 2010) and V8‐V9 using the V8f (Bradley et al., 2016) and 1510r (Amaral‐Zettler et al., 2009). Amplifications were performed in a reaction mixture containing ~5 ng of extracted DNA in master mix as mentioned above. PCR cycles were set at initial denaturation of 95°C for 300 s, followed by 10 cycles of denaturation at 95°C for 30 s, annealing at 47°C for 45 s, extension at 72°C for 60 s, and subsequently 15 cycles of denaturation at 95°C for 35 s, annealing at 57°C for 40 s, extension at 72°C for 60 s, with a final extension step of 10 min at 72°C for both markers. A negative control was included containing only nuclease free water. A field sample was included as a positive control. The amplified products were visualized on an agarose gel under UV illumination. The only aim of this step was to verify that these primers were also able to detect these species. No sequencing of the test community for short read amplicons was conducted.

### 2.4 Library preparation and sequencing

Library preparation was done following the manufacturer's guide for Native Barcoding Kit 24 V14 (SQK‐NBDD114.24, ONT, UK) with a few modifications for performance optimization. Briefly, 300 ng of the full‐length 18S PCR product (~1800 bp) of each sample was used as starting material for library preparation. The final library with ~25 fmol of DNA was loaded on the minION Spot‐on flowcell (FLO‐MIN114 version R10.4.1). Real‐time live basecalling was performed on the minION Mk1C via ont‐guppyd‐for‐mk1c v6.5.7 with the high accuracy option using default settings.

### 2.5 Basecalling models and accuracy

Basecalling was evaluated by the quality and accuracy of the resulting reads. Three basecalling methods were used to evaluate the accuracy and to determine which model would be the best fit (Table 1). The test community data were used to examine basecalling using the high‐accuracy model (HUP) using guppy and the super high accuracy (SUP) basecalling using dorado v0.5.0 (https://github.com/nanoporetech/dorado) with the dna_r10.4.1_e8.2_400bps_fast@v4.2.0 model for both simplex and duplex basecalling. The duplex option of the SUP model appeared to be vastly superior to the HUP model (Table 1). Dorado is optimized for GPU use; therefore, it is computationally more intense than HUP.

**TABLE 1:** Comparison of Nanopore MinION basecalling efficiency using three different models (super high accuracy [SUP‐duplex and SUP‐simplex] and high accuracy basically using guppy [HUP]).

Species	Models											
	SUP duplex				SUP simplex				HUP			
	% Similarity	Length (bp)	# mis‐matches	# gaps	% Similarity	Length (bp)	# mis‐matches	# gaps	% Similarity	Length (bp)	# mis‐matches	# gaps
*Dinophysis acuminata*	100	1743	0	0	99.774	1773	2	2	99.605	1773	5	2
*Mesodinium rubrum*	99.870	1544	2	0	99.870	1544	2	0	99.477	1530	6	2
*Teleaulax amphioxeia*	99.888	1782	1	1	99.383	1784	5	6	99.476	1718	6	3
*Skeletonema pseudocostatum*	99.886	1755	2	0	99.715	1757	2	3	99.432	1762	6	4
*Dunaliella tertiolecta*	100	1737	0	0	99.829	1758	2	1	99.722	1758	3	1
*Isochrysis galbana*	100	1752	0	0	99.718	1772	2	2	99.718	1770	4	1

*Note*: The first column is the percentage similarity followed by the length of the query sequence, the number of mismatches and number of gaps for each model.

### 2.6 Metabarcoding for short read sequencing using environmental DNA

The amplified V4 and V8‐V9 regions of the 18S rDNA genes were visualized on a gel and quantified using nanodrop. Equimolar concentrations of individual PCR products were pooled together for each marker and pooled samples were purified using DNA Purification SPRI Magnetic Beads (ABM, Canada). The purified library was sequenced on an Illumina MiSeq platform using v3 (2 × 300 bp).

### 2.7 Data analysis

#### 2.7.1 Taxonomic annotation of the full‐length 18S rDNA

FastQC was performed on the fastq sequences of the samples to evaluate the quality of the reads based on the Phred value (>15). The fastq sequences were then converted to fasta and the resulting sequences were BLASTed against the PR^2^ v5.0.0 database. Next, each fasta identifier was replaced with the taxonomic identifier in the PR^2^ reference database. The best BLAST hit against the PR^2^ database was used to classify each sequence, and a positive identification was defined as a match with ≥90% similarity and minimum 1000 bp to categorize to a major group. The results were grouped into kingdom/subkingdom/infrakingdom based on the taxonomic systematics following the World Register of Marine Species (Horton et al., 2017) to assess the protist diversity. To categorize the reads at genus level, the longest sequence having ≥95% similarity matching the reference sequence was retained.

#### 2.7.2 Taxonomic annotation of the short read sequences using environmental DNA

Fastq files for all six field samples were checked for quality control using FastQC. Paired end MiSeq reads of 2 × 300 bp were analyzed using mothur v1.39.0 (Schloss et al., 2009). Amplicon Sequence Variants (ASV) were subjected to BLAST analysis against the PR^2^ database (v5.0.0). Assignments were accepted only if the ASV similarity was ≥90% and query length was ≥70%. Metazoans were eliminated from the results.

## 3 RESULTS

### 3.1 Evaluation of primers and taxa validation in the test community

The full‐length 18S rDNA sequencing results were ~1829 bp. This expected length was obtained because the primers were selected to capture the V8‐V9 region completely. A total of ~100,000 sequences were generated, and all were readily identified at the species level. All the six species included in the test community were identified to species level (≥99% similarity) using the high accuracy basecalling and positively identified with Sanger sequencing.

**FIGURE 1:**
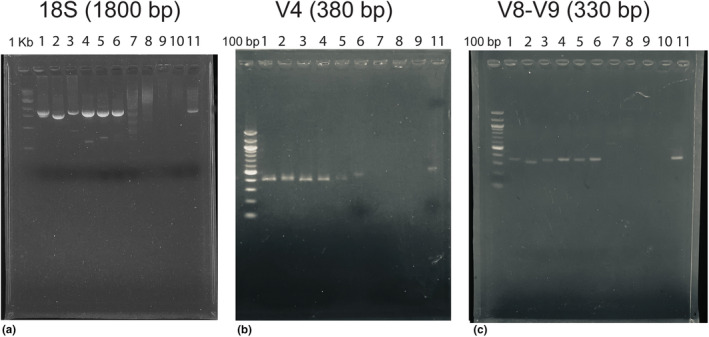
Gel electrophoresis of the PCR products amplified for the test community (a) 18S rDNA full‐length (~1800 bp), (b) V4 region of 18S rDNA (~380 bp), (c) V8‐V9 region of 18S rDNA gene (~330 bp). Lanes: 1‐ Dinoflagellate (*Dinophysis acuminata*); 2‐ Ciliate (*Mesodinium rubrum*); 3‐ Cryptophyte (*Teleaulax amphioxeia*); 4‐ Diatom (*Skeletonema pseudocostatum*); 5‐ Chlorophyte (*Dunaliella tertiolecta*); 6‐ Haptophyte (*Isochrysis galbana*); controls: 7‐ Cyanobacteria (*Synechococcus* sp.); 8‐ *E.coli*; 9‐ Blank; 10‐ Elution buffer blank; 11‐ field sample. Note the 18S rDNA PCR product of *Mesodinium* is shorter than the standard 1800 bp of the full‐length 18S rDNA (a).

Despite using equimolar DNA concentrations for the library preparation, the recovered read abundance was not evenly distributed across the species used in the test community. The sequence length of these ASVs were different (Figure 1). The 18S rDNA PCR product for *Mesodinium rubrum* was ~1600 bp compared to the ~1800 bp for the other species. The short read primers V4 and V8‐V9 also amplified all the species used in the test community.

### 3.2 Basecalling models and accuracy validation using the test community

Three different models were employed for basecalling of the Nanopore sequencing. Application of these models depends on the objective of the research. For example, if diversity at genus‐level is required, the HUP model provides the desired results as it is capable of basecalling at high accuracy of >99% (Table 1). From the comparison of the three models for the test community, it was evident that dorado duplex SUP basecalling provided super high accurate results at accuracy levels of 99.94% compared to 99.57% using HUP basecalling (Table 1). Although basecalling with dorado duplex SUP provides more accurate results than the HUP model, it is time consuming as well as computationally intense. The dorado duplex SUP basecalling is recommended for identification at the species level, especially in cases where harmful algal species must be differentiated from non‐harmful species.

### 3.3 Field samples

#### 3.3.1 Protist diversity using full‐length 18S rDNA

Nanopore sequencing of the six field DNA samples resulted in ~1.35 million reads. The high accuracy basecalling (HUP) using guppy produced sequences with Phred values of ~16. Based on the forced criteria of BLAST parameters of a minimum ≥90% similarity and ≥1000 bp, a total of 628,651 sequences (~47%) were taxonomically annotated. The remaining sequences shorter than 1000 bp were discarded, so focus remained on the remaining ~47% of the ASVs to assess protist group‐level or genus level classification.

**FIGURE 2:**
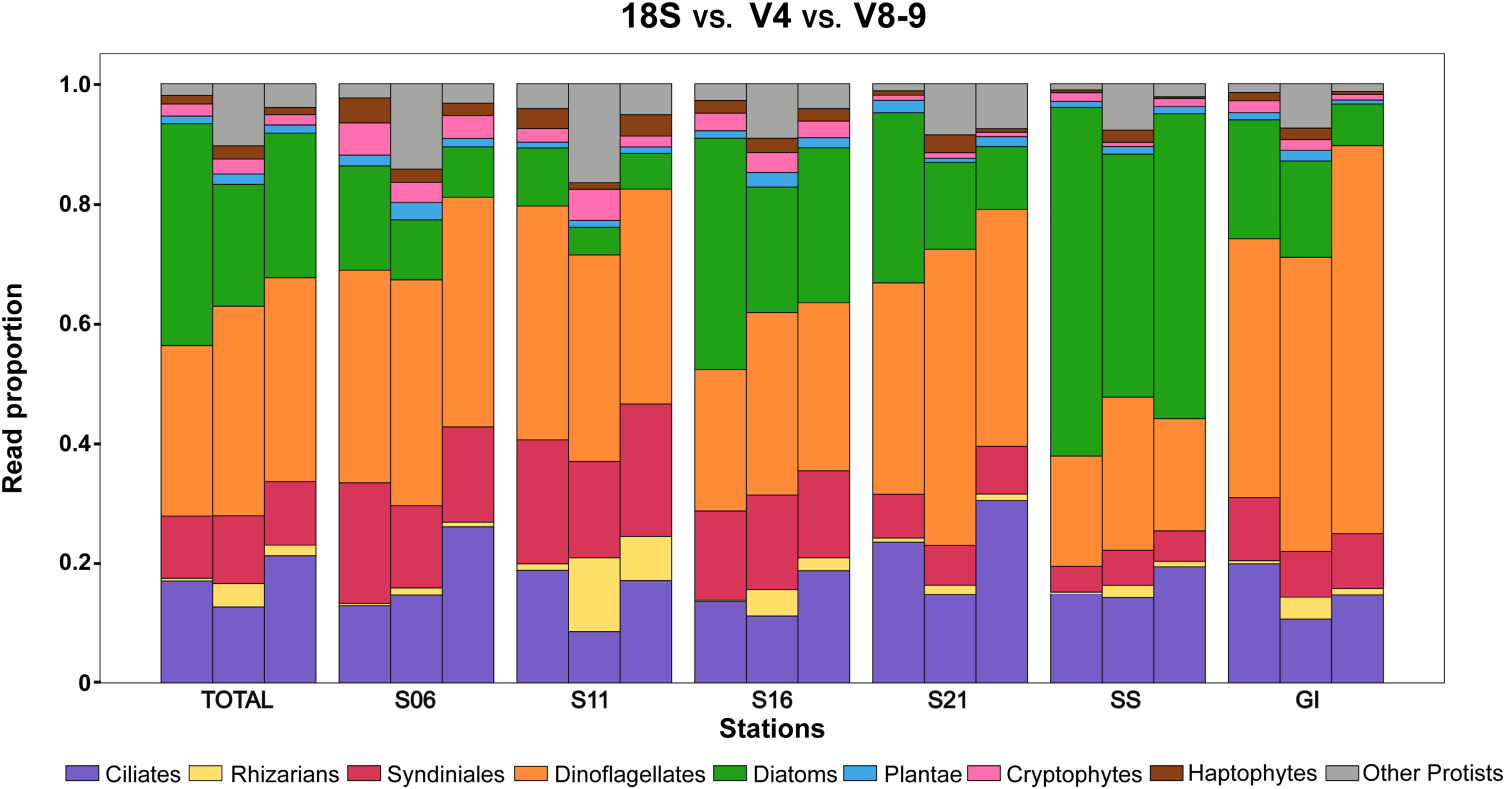
Field results for the relative read proportions of the total number of ASVs for full‐length 18S (left bar), V4 (center bar), and V8‐V9 (right bar) rDNA gene sequences associated with major protist groups, excluding the Opisthokonta (Campbell et al., 2024). Total for all stations and for each station along the Texas coast from south (S06) to northeast (GI) on 23 August 2017 (see Appendix 2 for station locations and read depth).

Overall, the protist community composition at the major group level revealed that diatoms and dinoflagellates were the dominant groups with all three markers (Figure 2). However, the community structure was variable among the three markers in individual samples (Figure 2). The V4 marker revealed a higher read proportion for the “other protist” group, and in most cases a lower diatom proportion. Dinoflagellates were dominant among the station GI sequences, while diatoms were dominant at station SS.

Based on the results from all markers, it is evident that not all the genera were detected by the set criteria (Table 2). A total of 298 genera were identified at ≥95% similarity match. Of these 298 genera observed by at least one of the three markers, the full‐length sequence revealed 250 genera, while MiSeq V4 and V8‐V9 identified only 226 and 213 genera, respectively (Table 2). Although diatoms were the most dominant based on ASV reads per genera, dinoflagellates were the most diverse, with 85 genera annotated in total (Appendices 3 and 4). Overall, the 18S sequencing allowed detection of almost all of the diatoms, ciliates, Plantae, cryptophytes, and radiolarians, but was less successful in identifying all cercozoans (only 50%) or dinoflagellates (78%) (Table 3).

**TABLE 2:** The number of the genera identified by the 18S, V4, and V8‐V9 rDNA gene markers.

	18S	V4	V8/9
**Dinoflagellates**			
*Akashiwo*			
*Alexandrium*			
*Amoebophrya*			
*Amphidinium*			
*Amphidoma*			
*Ansanella*			
*Archaeperidinium*			
*Azadinium*			
*Barrufeta*			
*Biecheleria*			
*Biecheleriopsis*			
*Blastodinium*			
*Blixaea*			
*Brachidinium*			
*Ceratoperidinium*			
*Chimonodinium*			
*Chytriodinium*			
*Dinophysis*			
*Diplopsalis*			
*Duboscquodinium*			
*Ensiculifera*			
*Euduboscquella*			
*Fragilidium*			
*Fusiperidinium*			
*Gertia*			
*Gonyaulax*			
*Grammatodinium*			
*Gymnodinium*			
*Gymnoxanthella*			
*Gyrodiniellum*			
*Gyrodinium*			
*Heterocapsa*			
*Ichthyodinium*			
*Islandinium*			
*Karenia*			
*Karlodinium*			
*Lepidodinium*			
*Lessardia*			
*Luciella*			
*Margalefidinium*			
*Naiadinium*			
*Noctiluca*			
*Oodinium*			
*Paragymnodinium*			
*Pelagodinium*			
*Pentapharsodinium*			
*Peridinium*			
*Phalachroma*			
*Polykrikos*			
*Posoniella*			
*Prorocentrum*			
*Proterythropsis*			
*Protodinium*			
*Protoperidinium*			
*Scrippsiella*			
*Shimiella*			
*Stoeckeria*			
*Syndinium*			
*Takayama*			
*Theleodinium*			
*Torodinium*			
*Torquentidium*			
*Tripos*			
*Wangodinium*			
*Warnowia*			
*Yihiella*			
*Zooxanthella*			
*Achradina*			
*Adenoides*			
*Apocalathium*			
*Balechina*			
*Blepharocysta*			
*Brandtodinium*			
*Cladocopium*			
*Cucumeridinium*			
*Durinskia*			
*Erythropsidinium*			
*Kapelodinium*			
*Levanderina*			
*Palatinus*			
*Parvodinium*			
*Podolampas*			
*Ptychodiscus*			
*Spiniferodinium*			
*Symbiodinium*			
**Diatoms**			
*Actinocyclus*			
*Actinoptychus*			
*Arcocellulus*			
*Asterionellopsis*			
*Asteromphalus*			
*Bacillaria*			
*Bacteriastrum*			
*Bacterosira*			
*Cerataulina*			
*Chaetoceros*			
*Coscinodiscus*			
*Cyclotella*			
*Cylindrotheca*			
*Cymbella*			
*Dactyliosolen*			
*Delphineis*			
*Ditylum*			
*Eucampia*			
*Fragilariopsis*			
*Guinardia*			
*Helicotheca*			
*Hemiaulus*			
*Lauderia*			
*Leptocylindrus*			
*Meuniera*			
*Minidiscus*			
*Minutocellus*			
*Navicula*			
*Nitzschia*			
*Odontella*			
*Papiliocellulus*			
*Planktoniella*			
*Pleurosigma*			
*Pseudo‐nitzschia*			
*Pseudosolenia*			
*Rhaphoneis*			
*Rhizosolenia*			
*Shionodiscus*			
*Skeletonema*			
*Stephanodiscus*			
*Synedra*			
*Tabularia*			
*Talaroneis*			
*Tenuicylindrus*			
*Thalassionema*			
*Thalassiosira*			
*Thalassiothrix*			
*Trieres*			
*Tryblionella*			
*Fragilaria*			
*Grammonema*			
*Haslea*			
**Ciliates**			
*Amphorellopsis*			
*Antestrombidium*			
*Apostrombidium*			
*Askenasia*			
*Bergeriella*			
*Codonellopsis*			
*Cyclotrichium*			
*Cyrtostrombidium*			
*Dartintinnus*			
*Didinium*			
*Ephelota*			
*Euplotes*			
*Eutintinnus*			
*Halodinium*			
*Helicostomella*			
*Hexasterias*			
*Laboea*			
*Leegaardiella*			
*Lynnella*			
*Mesodinium*			
*Metacylis*			
*Myxophyllum*			
*Paralembus*			
*Parallelostrombidium*			
*Parastrombidinopsis*			
*Parauronema*			
*Pelagostrobilidium*			
*Peritromus*			
*Philasterides*			
*Porpostoma*			
*Pseudocohnilembus*			
*Pseudotontonia*			
*Pseudovorticella*			
*Rimostrombidium*			
*Sinistrostrombidium*			
*Spirostrombidium*			
*Spirotontonia*			
*Stenosemella*			
*Strombidinopsis*			
*Strombidium*			
*Synophrya*			
*Tintinnidium*			
*Tintinnopsis*			
*Urotricha*			
*Vampyrophrya*			
*Varistrombidium*			
*Zoothamnium*			
*Cothurnia*			
*Epicarchesium*			
*Favella*			
*Pseudokeronopsis*			
**Plantae‐Chlorophyta**			
*Bathycoccus*			
*Chlamydomonas*			
*Chloropicon*			
*Chloroparvula*			
*Crustomastix*			
*Desmodesmus*			
*Halochlorococcum*			
*Halosphaera*			
*Mamiella*			
*Mantoniella*			
*Marsupiomonas*			
*Micromonas*			
*Monornaaphidium*			
*Nannochloris*			
*Nephroselmis*			
*Oltmannsiellopsis*			
*Ostreococcus*			
*Picochlorum*			
*Picocystis*			
*Prasinopapilla*			
*Pterosperma*			
*Pycnococcus*			
*Pyramimonas*			
*Scenedesmus*			
*Scherffelia*			
*Tetraselmis*			
*Ulva*			
*Prasinoderma*			
*Choricystis*			
*Pseudodidymocystis*			
*Prasinococcus*			
**Plantae‐Rhodophyta**			
*Neorhodella*			
*Dixoniella*			
**Haptophyta**			
*Algirosphaera*			
*Braarudosphaera*			
*Calyptrosphaera*			
*Chrysochromulina*			
*Cruciplacolithus*			
*Dicrateria*			
*Emiliania*			
*Gephyrocapsa*			
*Haptolina*			
*Isochrysis*			
*Pavlomulina*			
*Phaeocystis*			
*Prymnesium*			
*Syracosphaera*			
*Tisochrysis*			
*Chrysoculter*			
*Chrysotila*			
*Pseudohaptolina*			
*Reticulofenestra*			
**Cryptophytes**			
*Chroomonas*			
*Geminigera*			
*Goniomonas*			
*Hemiselmis*			
*Proteomonas*			
*Rhodomonas*			
*Storeatula*			
*Teleaulax*			
*Kathablepharis*			
*Leucocryptos*			
*Hemiarma*			
*Urgorri*			
**Cercozoa**			
*Cryothecomonas*			
*Lotharella*			
*Mataza*			
*Paulinella*			
*Phagomyxa*			
*Protaspa*			
*Abollifer*			
*Minorisa*			
*Peregrinia*			
*Reckertia*			
*Thaumatomastix*			
*Ventrifissura*			
**Radiolaria**			
*Acanthometra*			
*Amphibelone*			
*Haliommatidium*			
*Protoscenium*			
*Stauracantha*			
**Other Protists (Amoebozoa, Euglenozoa)**			
*Vermistella*			
*Paramoeba*			
*Ophirina*			
*Lacrimia*			
**Other Ochrophyta (Pelagophytes, Raphidophytes, etc)**			
*Ankylochrysis*			
*Apedinella*			
*Chattonella*			
*Fibrocapsa*			
*Haramonas*			
*Heterosigma*			
*Lepidochromonas*			
*Nannochloropsis*			
*Paraphysomonas*			
*Phaeomonas*			
*Pseudopedinella*			
*Rhizochromulina*			
*Triparma*			
*Pedinella*			
**Other Chromista (Bigyra, Oomycota, Protozoa)**			
*Aplanochytrium*			
*Bicosoeca*			
*Caecitellus*			
*Pseudobodo*			
*Cafileria*			
*Oblongichytrium*			
*Lagenidium*			
*Pirsonia*			
**Other Alveolata (Apicomplexa, Chrompodellids)**			
*Cyclospora*			
*Goussia*			
*Alphamonas*			

*Note*: Blue shading indicates positive identification; white shading indicates the marker did not detect the genus.

**TABLE 3:** The number of genera identified with either full length 18S, V4, V8‐V9, or all three markers for Dinoflagellates (Dino), Diatoms, Ciliates, Chlorophyta and Rhodophyta (as Plantae), Haptophyta (Hapto), Cryptophyta (Crypto), Cercozoa (Cerco), Radiolaria (Radio) and all other Protozoans, Discoba, Apicomplexa, Raphidophytes and non‐photosynthetic single‐cell genera (as Other Protists).

Detected by	Dino (85)	Diatoms (52)	Ciliates (51)	Plantae (33)	Hapto (19)	Crypto (12)	Cerco (12)	Radio (5)	Other protists (29)	Total %
18S	66	49	47	30	15	10	6	4	22	84
V4	66	38	36	28	12	10	11	3	22	76
V8/9	54	47	36	19	14	8	7	5	25	71
All 3 markers	37	30	21	16	8	7	4	3	17	48

*Note*: Of the total 298 genera identified, the 18S results captured the largest percentage.

Among the diatoms, based on the full‐length 18S Nanopore data, *Cyclotella* was the most abundant based on number of ASVs, followed by *Thalassiosira* and *Chaetoceros* (Appendix 3). For the V4 marker, *Thalassiosira* had the most ASVs, followed by *Cyclotella* and *Chaetoceros*, whereas based on the V8‐V9 marker *Cyclotella* had the most ASVs, followed by *Thalassiosira* and *Chaetoceros*. Among the dinoflagellates, in the full‐length 18S data, *Gyrodinium* had the highest number of ASVs, followed by *Protoperidinium* and *Heterocapsa*. Based on the V4 marker, *Ptychodiscus* was the most dominant, but this genus was only observed with the V4 marker and not in the results with either of the other two markers even at 95%. *Gyrodinium* and *Torodinium* followed *Ptychodiscus*, whereas with the V8‐V9 marker, *Karenia* had the highest number of ASVs, followed by *Ensiculifera* and *Gyrodinium* (Appendix 4).

## 4 DISCUSSION

Assessing biodiversity using the rDNA markers has become an essential tool for the study of protist communities in aquatic ecosystems. However, the lack of universal primers for selected markers can lead to the limited coverage of species diversity. Here a protocol is presented for amplification of the full‐length 18S rDNA (~1800 bp) using a new combination of primers (SSUF and ITS‐1dr), followed by sequencing and analysis using the ONT minION. The protocol for Nanopore sequencing, from library preparation to the start of sequencing, is relatively simple and was performed in less than 15 min. The sequencing depth should be determined by the objective of the study. In this study, a good sampling depth was considered by obtaining ~100,000 raw read sequences per sample (Appendix 2). This coverage was considered optimum for this case, as it was possible to include even the very low abundant taxa. Also, with the online basecalling provided on the minION the user can visualize the amount of sequence reads generated for each sample, as well as the quality of the sequences, in real‐time and can terminate the sequencing run once the number of sequences generated for the samples is satisfactory. The flowcell can be cleaned and subsequently used to load a new library, if the pores have not deteriorated. An additional benefit of the real‐time data generated using the minION is that species detection can be in near‐real‐time. Aquaculture industries in particular need real‐time data for detecting the presence of harmful species.

To evaluate the proposed protocol for assessing protist community diversity, the efficiency of the full‐length 18S metabarcode, the V4 and V8‐V9 short read primer pairs were first compared in a test community. All three marker primer sets successfully amplified all the taxa used in the test community. The taxa used in the test community did show different read counts; however, this is likely attributed to differences in ribosomal copy numbers among the different taxa. Even optimizing the DNA concentration used for library preparation can produce different read counts because of the composition of the DNA (Thermo Scientific, 2009).

The new primer combination was successful in detecting 84% of the total taxonomically defined genera in the field samples. A reason for the lack of amplification of all taxa in the field data can be primer misfit, which can lead to discriminatory amplification of certain taxa over others (Liu et al., 2023). This was the case for dinoflagellate *Achradina* which had 7 mismatches in the binding site of the forward primer. Even though the reverse primer matched, the result was not a full‐length 18S rDNA sequence. The presence of introns in primer sites can also limit the taxa detection (Gaonkar et al., 2018). With the V4 marker, the presence of an intron in the V4 region leads to longer fragments and lowers the overlap percentage and hence the sequence is excluded in the pre‐cleaning steps (Gaonkar et al., 2020). Another important variable can be bias in primer binding affinity to all taxa in the field samples. Additionally, sub‐optimal field sampling, sample handling, DNA extractions, bioinformatic procedures, including the use of incomplete or uncurated reference sequence databases followed by sequencing errors are also potential fundamental mistakes in metabarcoding studies (Marinchel et al., 2023; Ruppert et al., 2019).

In other cases, taxa can be missed. An explanation for the lack of taxa detection is the unavailability of the full‐length reference sequences which can lead to reduction in estimation of species diversity. In some instances, the standard 18S rDNA reverse primer region does not overlap the V9 region (Gaonkar et al., 2018), so the short read V9 ASV may not match its exact reference sequence. For example, the absence of *Ptychodiscus* in the 18S and V8‐V9 results (Table 2) may be related to the lack of full‐length 18S rDNA reference in GenBank (*Ptychodiscus* KU640194). Although there were 18S sequences that matched *Ptychodiscus*, they were only 1446 bp with 92.393% similarity, so were removed from analysis because they did not meet our established criteria for full length. Another reason for the absence of some taxa in the sample would be a very low cell abundance at the time of collection. Some examples include the ciliate *Favella* or the diatom *Haslea*, which were present at low abundance in the field samples (Campbell & Henrichs, 2020) and ASVs were not found in the full‐length 18S results.

One of the important resources needed for successful DNA metabarcoding is the availability of full‐length taxonomically validated reference databases. Most of the reference sequences available are for the most extensively studied genera, whereas the small <5‐micron algae (many of which are small dinoflagellates) are not as well characterized, so are unavailable in the reference database. A total of 19 dinoflagellate genera were not detected using the full‐length 18S sequences, compared to only three in diatoms.

Although the minION produces an enormous amount of sequence data, the absence of a proper taxonomically validated reference database becomes the bottleneck for taxa identification. If the research objective is to assess or analyze to the class or genus level, then the available PR^2^ v5.0.0 reference database satisfies the requirement. However, there are a large number of ASVs in the field data presented here that are not characterized at genus level or even at the class level. These unclassified ASVs can be included in the group totals in determining the total protist community structure but cannot be used in detailed studies of protist diversity. Consequently, among the ~47% of the ASVs that were taxonomically annotated to a class or family, only ~28% of the dinoflagellates could be taxonomically identified to genus level compared to 96% of the diatoms. Likely this observation is because diatoms are more extensively studied compared to dinoflagellates. But if the requirements are to study the total species diversity within a genus, then a highly curated taxonomic database for species becomes a critical requirement. For example, even for the widely studied diatom genus *Chaetoceros* only 48% of the species can be identified with full‐length 18S (Gaonkar et al., 2018), which highlights the importance of having a taxonomically validated reference sequence database.

It appears that defining the protist community based only on short read ASVs could also tend to overestimate diversity if using BLAST alone. BLAST analysis alone may not be helpful in such cases, so a phylogenetic approach should be employed. This is recommended because short read ASVs associated with one species when clustered can map against multiple species. For example, the genetic difference between all the six known species within the toxic dinoflagellate *Karenia* is 1.6% (i.e., 28 bp in 1729 bp). When a consensus sequence of all the six species of *Karenia* is aligned with two additional species of *Karlodinium*, the result is 14 variable sites among the 1685 bp positions (i.e., 0.8% genetic difference). This information is valuable to know what to expect from the metabarcoding data, especially when differentiating non‐harmful species from harmful species in monitoring programs. Validation using phylogenies is necessary because of the low resolution of the 18S rDNA genes among closely related genera (e.g., *Karenia*, *Karlodinium*). Since the difference is relatively small in the short reads, ASVs can overestimate the taxa diversity if relying solely on BLAST, especially in the toxic dinoflagellate *Karenia* (Campbell et al., 2022; Gaonkar & Campbell, 2023).

Other examples include the *Fragilaria*/*Fragilariopsis*/*Grammonema* complex which are identical, so that the short reads matched all three genera, while the long read matched the original *Fragilariopsis* sequence only. This may also explain why some genera were only detected with V4 or V8‐V9 and not 18S, if the reference database is incomplete.

Genetic data can be considered equivalent to microscopes for marine molecular ecologists involved in investigating protist diversity in the environment. The advent of Nanopore sequence technology has provided the tools for long read sequencing cost effectively. Moreover, it is important to note that the new full‐length 18S primer combination generates longer amplicons, which will allow for more precise species delineation and protist diversity than the short read V4 or V8‐V9 markers. Overall, in using metabarcoding one needs to consider multiple technical aspects (e.g. Elbrecht et al., 2017), but the methodology proposed here can be considered an update for studying protist community structure and diversity. Obtaining full‐length 18S rDNA sequences from environmental samples can fill the gaps that exist in the current reference sequences. In summary, this work demonstrates that the full‐length 18S rDNA metabarcoding approach using Nanopore can be successfully applied for investigating protist diversity in field studies.

## AUTHOR CONTRIBUTIONS

**Chetan C. Gaonkar:** Conceptualization (equal); data curation (equal); formal analysis (lead); methodology (lead); project administration (equal); validation (equal); visualization (lead); writing – original draft (equal); writing – review and editing (equal). **Lisa Campbell:** Conceptualization (equal); data curation (equal); formal analysis (supporting); funding acquisition (lead); investigation (equal); methodology (supporting); project administration (equal); resources (lead); supervision (lead); validation (equal); visualization (supporting); writing – original draft (equal); writing – review and editing (equal).

## CONFLICT OF INTEREST STATEMENT

The authors have no conflict of interest to declare.

## BENEFIT‐SHARING STATEMENT

The data that support the findings of this study are openly available in GenBank. Benefits from this research accrue from the sharing of our data and results on public databases as described above in Data Accessibility statement.

## Data Availability

The data that support the findings of this study are openly available as .fastq files in NCBI's SRA archive under accession number PRJNA592369 (https://www.ncbi.nlm.nih.gov/bioproject/PRJNA592369). The taxonomic annotations for individual sequences are available in the Biological and Chemical Oceanography Data Management Office database (https://www.bco‐dmo.org/project/715170).

## APPENDIX 1 ISOLATION INFORMATION AND CULTURE CONDITIONS FOR THE SPECIES USED IN THE TEST COMMUNITY TO VALIDATE NEW PRIMER COMBINATION.

**Table:**

Category	Species	Strain ID	Collected by	Collection date	Geographic location	Media	Salinity	GenBank match at 100% similarity
Chlorophyte	*Dunaliella tertiolecta*	CCAP 19/30	Unknown	2016	Unknown	f/2	35	MW471050
Ciliate	*Mesodinium rubrum*	MBL‐DK2009	P. J. Hansen	2009	Helsingør Harbor, Denmark	L1	22	AJ506972
Cryptophyte	*Teleaulax amphioxeia*	K‐0434	D. Hill	2009	The Sound, Denmark	L1	22	AJ421146
Diatom	*Skeletonema pseudocostatum*		D. W. Henrichs	2010	Surfside Beach, TX, USA	f/2	35	AJ632207
Dinoflagellate	*Dinophysis acuminata*	DAVA01	J. M. Fiorendino	2019	Surfside Beach, TX, USA	L1	22	AJ506972
Haptophyte	*Isochrysis galbana*	CCMP1323	M. Parke	1938	Port Erin, Isle of Man	f/2	35	DQ079859

## APPENDIX 2 FIELD SAMPLE COLLECTION LOCATIONS AND THE SEQUENCING DEPTH FOR EACH MARKER.

**Table:**

Station	Lat/Long	18S	V4	V8‐V9
Galveston Island (GI)	29.0649 N/94.9000 W	170,643	316,377	667,272
Surfside (SS)	28.9600 N/95.0946 W	394,223	327,278	333,885
S21	28.7644 N/95.2978 W	197,131	341,298	456,412
S16	28.5366 N/95.8656 W	244,160	428,515	354,632
S11	28.2614 N/96.4129 W	228,918	430,498	399,193
S06	27.8358 N/96.9874 W	109,706	422,857	367,599

## APPENDIX 3 COMPARISON OF ASV READ ABUNDANCE OF DIATOM GENERA IN 18S FULL LENGTH, V4 AND V8‐V9 METABARCODING DATA.

**Table:**

Genus	ASV (≥95%)	Reads (≥95%)	L1_S06_V9	L1_S11_V9	L1_S16_V9	L1_S21_V9	L1_SS_V9	L1_GI_V9
*Actinocyclus*	30	63	5	3	5	20	15	15
*Actinoptychus*	3	3	0	2	0	1	0	0
*Asterionellopsis*	57	167	1	0	4	136	21	5
*Bacillaria*	6	7	0	0	3	1	2	1
*Bacteriastrum*	51	60	0	0	25	12	0	23
*Chaetoceros*	2928	9567	771	288	1193	2066	4916	333
*Coscinodiscus*	142	389	160	128	10	17	6	68
*Cyclotella*	4392	31,091	26	167	5036	1207	23,463	1192
*Cylindrotheca*	432	1643	12	60	459	132	423	557
*Cymbella*	40	130	0	6	66	12	36	10
*Ditylum*	56	163	32	3	1	49	54	24
*Eucampia*	85	200	8	1	3	84	84	20
*Fragilaria*	30	60	6	7	12	25	7	3
*Fragilariopsis*	23	39	2	2	8	11	6	10
*Grammonema*	24	40	0	3	11	5	15	6
*Guinardia*	1207	4883	161	59	696	2715	1111	141
*Haslea*	9	16	1	0	1	8	6	0
*Helicotheca*	5	12	4	0	0	2	4	2
*Hemiaulus*	41	58	7	0	3	26	21	1
*Lauderia*	31	88	6	0	2	37	35	8
*Leptocylindrus*	535	1566	60	17	126	830	520	13
*Minidiscus*	307	835	3	12	265	50	394	111
*Minutocellus*	477	1555	10	26	451	38	898	132
*Navucula*	13	13	0	0	6	2	3	2
*Nitzschia*	210	623	5	43	160	92	90	233
*Odontella*	296	976	190	9	0	238	503	36
*Opephora*	25	41	4	2	3	13	18	1
*Papiliocellulus*	15	16	0	0	7	1	7	1
*Planktoniella*	27	74	0	2	12	1	58	1
*Pleurosigma*	74	168	12	2	7	113	22	12
*Pseudo‐nitzschia*	1224	4531	558	75	266	2644	854	134
*Pseudosolenia*	38	131	64	1	0	46	17	3
*Rhizosolenia*	48	146	45	11	3	34	15	38
*Shionodiscus*	56	108	0	0	6	3	99	0
*Skeletonema*	1051	3646	154	124	1658	296	1161	253
*Stephanodiscus*	19	28	0	0	11	3	12	2
*Synedra*	3	4	0	1	0	2	1	0
*Tabularia*	1	1	0	0	0	1	0	0
*Tenuicylindrus*	307	1015	1	1	225	51	728	9
*Thalassionema*	36	130	15	1	15	59	18	22
*Thalassiosira*	4278	23,832	455	1229	11,382	2372	7681	713
*Thalassiothrix*	139	427	251	27	2	120	22	5
*Trieres*	2	2	0	0	1	0	1	0
*Tryblionella*	9	9	2	0	2	0	5	0

## APPENDIX 4 COMPARISON OF ASV READ ABUNDANCE OF DINOFLAGELLATE GENERA IN 18S FULL LENGTH, V4 AND V8‐V9 METABARCODING DATA.

**Table:**

Genus 18S	Total ASVs (>95)	18S_SO6	18S_S11	18S_S16	18S_S21	18S_SS	18S_GI
*Akashiwo*	4	0	0	0	0	3	1
*Alexandrium*	258	4	18	29	42	53	112
*Amphidinium*	398	0	21	22	76	229	50
*Amphidoma*	176	2	17	30	15	8	104
*Ankistrodinium*	1	0	0	0	0	1	0
*Ansanella*	16	0	0	1	0	11	4
*Archaeperidinium*	87	41	25	2	7	12	0
*Azadinium*	52	2	12	5	8	6	19
*Barrufeta*	103	13	3	0	20	66	1
*Biecheleria*	272	0	5	98	8	75	86
*Biecheleriopsis*	136	0	28	37	8	9	54
*Blastodinium*	9	0	9	0	0	0	0
*Blixaea*	34	29	2	3	0	0	0
*Ceratium*	11	1	0	0	0	0	10
*Chimonodinium*	2	0	0	0	0	2	0
*Chytriodinium*	66	2	35	22	5	1	1
*Dinophysis*	2	0	0	0	0	2	0
*Diplopsalis*	22	5	11	0	6	0	0
*Duboscquodinium*	402	95	47	32	24	27	177
*Ensiculifera*	4	0	0	0	0	3	1
*Fragilidium*	17	17	0	0	0	0	0
*Gertia*	16	0	1	5	2	2	6
*Gonyaulax*	1316	596	649	27	25	5	14
*Grammatodinium*	82	0	0	16	5	61	0
*Gymnodinium*	657	63	89	152	52	S	301
*Gymnoxanthella*	68	0	2	17	10	24	15
*Gyrodiniellum*	7	2	1	2	0	0	2
*Gyrodinium*	15,556	1305	2208	2135	3275	4486	2147
*Heterocapsa*	3299	508	603	476	551	544	617
*Islandinium*	93	1	2	0	29	55	6
*Karenia*	705	5	109	44	27	14	506
*Karlodinium*	2233	37	639	300	312	244	701
*Kryptoperidinium*	2	0	2	0	0	0	0
*Leiocephalium*	2	0	1	0	0	1	0
*Lepidodinium*	1071	69	115	194	291	178	224
*Lessardia*	23	0	0	5	2	4	12
*Levanderina*	2	0	0	0	0	2	0
*Luciella*	2	0	0	0	0	2	0
*Margalefidinium*	215	49	3	6	59	52	46
*Naiadinium*	1	1	0	0	0	0	0
*Nematodinium*	2	0	0	0	0	1	1
*Noctiluca*	24	24	0	0	0	0	0
*Oodinium*	1012	0	489	184	0	129	210
*Oodinium*	6	6	0	0	0	0	0
*Paragymnodinium*	178	7	20	40	17	88	6
*Pelagodinium*	279	1	113	74	0	37	54
*Pentapharsodinium*	174	6	23	34	34	42	35
*Peridinium*	55	3	12	11	7	20	2
*Phalachroma*	46	0	18	0	27	0	1
*Polarella*	1	0	0	0	0	0	1
*Polykrikos*	84	51	1	0	11	7	14
*Posoniella*	1	1	0	0	0	0	0
*Prorocentrum*	1599	118	329	378	199	209	366
*Proterythropsis*	20	11	0	0	9	0	0
*Protodinium*	3	1	0	0	0	0	2
*Protoperidinium*	4029	235	232	420	449	2333	360
*Scrippsiella*	1020	48	94	190	99	258	331
*Spiniferites*	16	0	0	0	8	4	4
*Stoeckeria*	117	9	37	32	4	31	4
*Syltodinium*	6	0	0	1	1	4	0
*Takayama*	3214	29	74	432	574	287	1818
*Theleodinium*	1	0	0	0	0	1	0
*Torodinium*	8	0	4	0	1	2	1
*Torquentidium*	1233	175	651	31	196	91	89
*Tripos*	492	96	136	0	51	28	181
*Wangodinium*	14	0	1	1	1	5	6
*Warnowia*	4	1	0	0	1	0	2
*Yihiella*	3	0	1	0	0	1	1
